# Higher critical closing pressure is independently associated with enlarged basal ganglia perivascular spaces

**DOI:** 10.3389/fneur.2023.1165469

**Published:** 2023-10-05

**Authors:** Jiayi Zhong, Wanrong Lin, Junru Chen, Qingchun Gao

**Affiliations:** Department of Neurology, The Second Affiliated Hospital of Guangzhou Medical University, Guangzhou, China

**Keywords:** cerebral small vessel diseases, enlarged perivascular spaces, critical closing pressure, cerebral autoregulation, cerebral hemodynamic

## Abstract

**Objective:**

This study aimed to explore the association between cerebral hemodynamic parameters focused on the critical closing pressure (CCP) and enlarged perivascular spaces (EPVS).

**Methods:**

Cerebral blood velocity in the middle cerebral artery (MCAv) and non-invasive continuous blood pressure (NIBP) were measured using a transcranial Doppler (TCD) and Finometer, followed by the calculation of cerebral hemodynamic parameters including CCP, resistance area product (RAP), pulsatility index (PI), and pulse pressure (PP). EPVS were graded separately in the basal ganglia (BG) and centrum semiovale (CSO), using a visual semiquantitative ordinal scale. Patients with EPVS >10 were classified into the severe BG-EPVS group and severe CSO-EPVS group, and the remainder into the mild BG-EPVS group and the mild CSO-EPVS group. Spearman’s correlation and binary logistic regression analysis were performed to analyze the relationship between hemodynamic parameters and BG-EPVS and CSO-EPVS, respectively.

**Results:**

Overall, 107 patients were enrolled. The severe BG-EPVS group had higher CCP, mean arterial blood pressure (MABP), systolic blood pressure (SBP), and diastolic blood pressure (DBP) than that in the mild BG-EPVS group (*p* < 0.05). There was no statistical difference in hemodynamic parameters between the severe CSO-EPVS group and the mild CSO-EPVS group. Spearman’s correlation analysis showed that CCP was positively associated with BG-EPVS (*rho* = 0.331, *p* < 0.001) and CSO-EPVS (*rho* = 0.154, *p* = 0.044). The binary logistic regression analysis showed that CCP was independently associated with severe BG-EPVS (*p* < 0.05) and not with CSO-EPVS (*p* > 0.05) after adjusting for confounders.

**Conclusion:**

CCP representing cerebrovascular tension was independently associated with BG-EPVS.

## 1. Introduction

Perivascular spaces, also known as the Virchow–Robin spaces, are compartments that surround the perforating arterioles and venules as they travel through the brain parenchyma from the subarachnoid space and function as protolymphatic systems that aid in the clearance of interstitial fluid and solutes (1). Enlarged perivascular spaces (EPVS) can be detected and quantified using brain MRI, and are mainly observed in the basal ganglia (BG) and the centrum semioval (CSO) (2). EPVS were previously considered a normal neuroradiologic variant (3) and have recently emerged as subclinical markers of risk for cognitive impairment, dementia, and stroke (4–6). They have been considered as an MRI marker of cerebral small vessel diseases (CSVD) (7). Therefore, it is crucial to understand the pathogenesis and risk factors for EPVS.

Numerous recent studies have underlined the importance of cerebral vascular hemodynamics in the pathophysiological mechanisms of EPVS, specifically pulsatility index (PI) (8–11), cerebrovascular reactivity (CVR) (12, 13), and cerebral blood flow (CBF) (14). However, no previous clinical studies have examined associations between perivascular spaces and cerebral vascular tone.

Critical closing pressure (CCP) is an index of cerebral vascular tone, defined as the lowest arterial blood pressure at which vessels collapse and flow ceases, reflecting the degree of contraction and diastole of vascular smooth muscle (15, 16). CCP is also an important cerebral hemodynamic parameter for describing and quantifying the characteristics of the cerebrovascular bed in more detail. When cerebral autoregulation (CA) is intact, the microvascular bed responds to blood pressure pulses from the large arteries by raising or lowering CCP (17–19). Autoregulation describes the response of the cerebral vasculature to changes in perfusion pressure, through which cerebral blood flow is continuously regulated to maintain neural function (20). CCP can be assessed noninvasively through transcranial Doppler (TCD) by comparing the pulsatile waveforms of blood flow velocity and arterial blood pressure (21). Hsu HY et al. found a correlation between CCP and PI (22). However, no studies to date have investigated associations between CCP, PI, and EPVS.

Therefore, this study was conducted to explore the relationship between cerebral hemodynamic parameters focused on CCP, PI, and EPVS.

## 2. Methods

### 2.1. Patients

Between February 2022 and December 2022, this study collected data on 125 consecutive CSVD patients without acute cerebrovascular events who were admitted to the Neurology Department of the Second Affiliated Hospital of Guangzhou Medical University.

The inclusion criteria were as follows: 1. aged ≥45, 2. patients with CSVD evaluated by MRI in accordance with the guidelines for reporting vascular abnormalities on neuroimaging (STRIVE) (7). The exclusion criteria were as follows: 1. bilateral poor temporal window on TCD; 2. hypertension not under control or atrial fibrillation; 3. acute ischemic stroke, history of traumatic brain injury, severe stroke, or progressive neurological disease; 4. ultrasound of the carotid artery, TCD, magnetic resonance angiography (MRA), computed tomography angiography (CTA), or digital subtraction angiography (DSA) detected greater than 50% stenosis in intracranial or extracranial arteries; 5. presence of Parkinson’s disease, Parkinson’s plus syndromes, dementia, or any other neurodegenerative disease; 6. moderate to severe chronic respiratory disease or symptomatic cardiac failure; 7. claustrophobia, or unable to lie flat in the supine position for scanning.

### 2.2. Ethical standards statement

Ethical approval for this study was granted by the Clinical Research and Application Ethics Committee of the Second Hospital of Guangzhou Medical University according to the ethical standards outlined in the Declaration of Helsinki. Written informed consent was acquired from all participants or their authorized representatives before enrollment.

### 2.3. Demographics and clinical assessments

On admission, experienced neurologists conducted face-to-face consultations to collect information. General demographic information (including age and gender), vascular risk factors (smoking or drinking, body mass index [BMI], hypertension, diabetes mellitus, cerebrovascular disease), laboratory tests (including homocysteine, fasting blood glucose, hemoglobin A1c [HbA1c], total cholesterol, low-density lipoprotein, triglyceride, and hematocrit) were acquired. Smoking was the history of ever smoking (yes/no). Drinking status was divided into drinking and never drinking. Hypertension was defined as a history of hypertension, taking antihypertensive treatment, or documented elevated blood pressure (systolic ≥140 or diastolic ≥90 mmHg). Diabetes mellitus was defined by self-report or history of treatment for diabetes with diet, insulin, or oral hypoglycemic agents (23, 24). Cerebrovascular disease was defined as a self-reported stroke or transient ischemic attack (TIA), ascertained by a questionnaire and confirmed by medical records. Blood samples were obtained from each patient following an overnight fasting period for the measurement of homocysteine, hematocrit, lipids, glucose, and HbA1c.

### 2.4. Brain MRI acquisition and visual assessment of WMH and lacunes

MR imaging of the brain for all subjects was performed on a 1.5 T MRI Achieva scanner (Philips, Best, Netherlands) equipped with an 8-channel phased array head coil. Sequences included axial diffusion-weighted imaging (DWI), T1-weighted, T2-weighted, and fluid-attenuated inversion recovery (FLAIR). The following parameters were applied to DWI (echo time [TE], 90; repetition time [TR], 3,200), T2-weighted (TR 8,000; TE 102), FLAIR (TR 8,000; TE 102), and T1-weighted sequences (TR 500; TE 8.5): field of view (FOV), 250 × 250 mm; matrix size, 256 × 256; slice thickness, 5 mm; interslice gap, 1 mm.

Structural image analysis of CSVD features, including WMH, lacunes, and EPVS, was performed according to the STRIVE criteria (7). All MRI were independently evaluated by two experienced neurologists blinded to participant demographic and clinical data; any discrepancies were reevaluated at a consensus meeting. In detail, imaging features were scored as follows: deep and periventricular WMH were visually scored according to the Fazekas scale from 0 to 3, and their scores were summed up as a total Fazekas score between 0 and 6 (25) and the presence of lacunes (0 or 1) (7).

### 2.5. Visual assessment of EPVS

EPVS were defined as ≤2 mm round or linear cerebrospinal fluid-isointense lesions (T2 hyperintense and T1/FLAIR-hypointense with respect to the brain) along the course of penetrating arteries (26). EPVS detected on axial T2-weighted images were rated on the slice with the greatest number of EPVS, in both the BG and CSO, using a validated, semiquantitative ordinal scale from 0 to 3: 0 (no visible EPVS), 1 (1–5 EPVS), 2 (6–10 EPVS), or 3 (> 10 EPVS) for each hemisphere (27). The hemisphere with the higher score was used where hemispheres were asymmetric.

**Figure 1:**
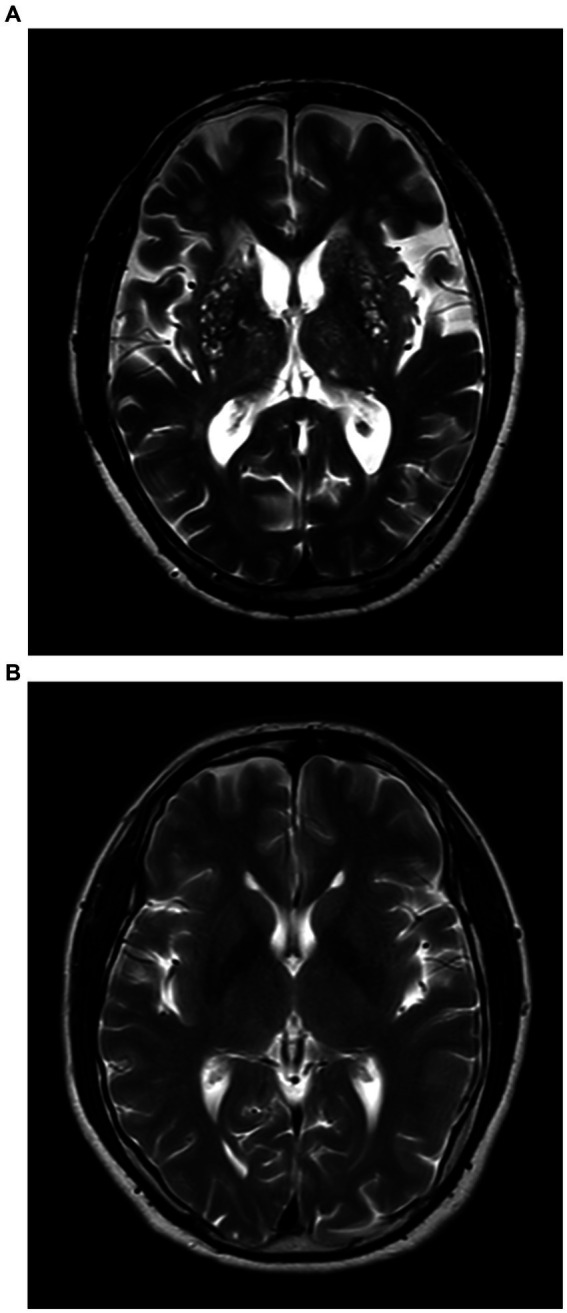
The severe enlarged perivascular spaces (EPVS) group and the mild EPVS group in the region of the basal ganglia (BG). **(A)** Patients with EPVS >10 in the region of the basal ganglia were defined as the severe BG-EPVS group; **(B)** Patients with EPVS ≤10 in the region of the basal ganglia were defined as the mild BG-EPVS group.

**Figure 2:**
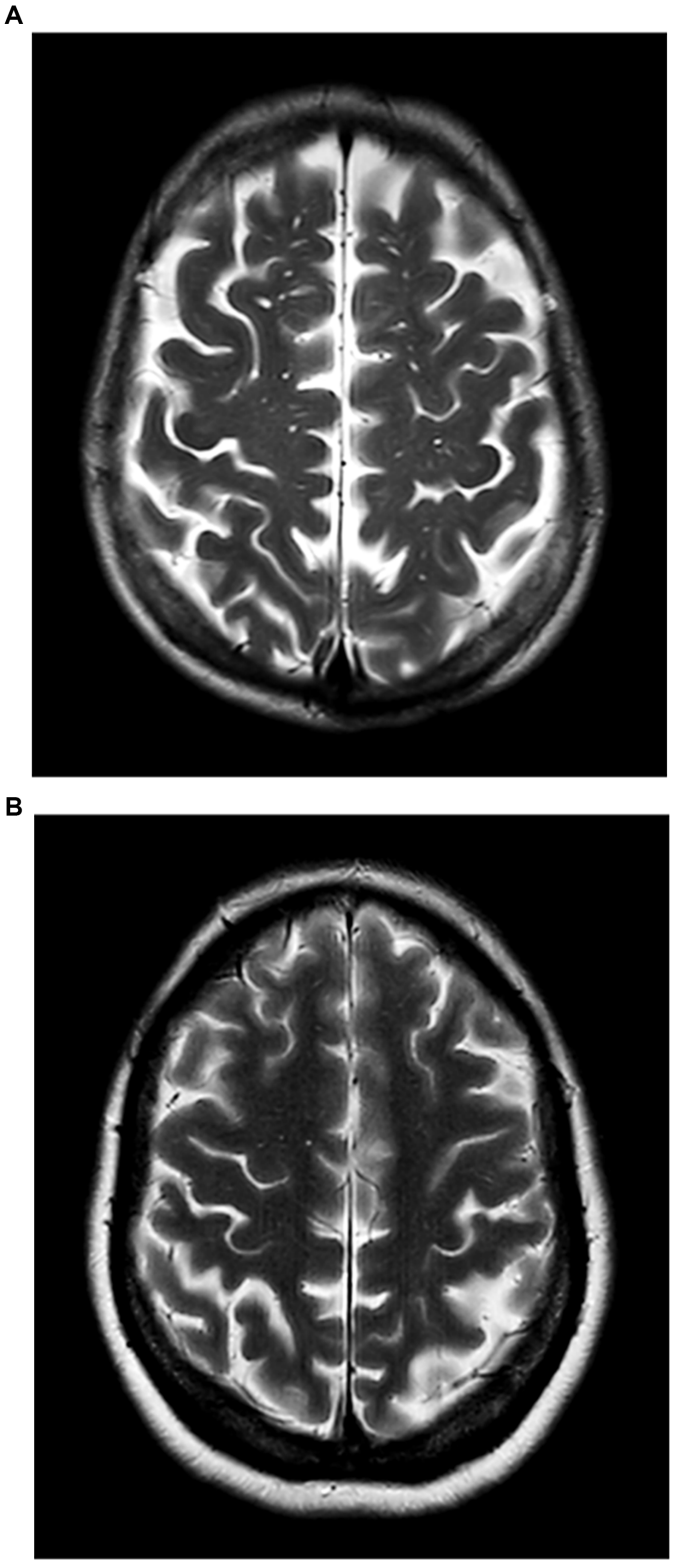
The severe enlarged perivascular spaces (EPVS) group and the mild EPVS group in the region of the centrum semiovale (CSO). **(A)** Patients with EPVS >10 in the region of the centrum semiovale were defined as the severe CSO-EPVS group; **(B)** Patients with EPVS ≤10 in the region of the centrum semiovale were defined as the mild CSO-EPVS group.

Patients with BG-EPVS >10 were classified into the severe BG-EPVS group (Figure 1A) and the remainder in the mild group (BG-EPVS ≤10) (Figure 1B). Patients with CSO-EPVS >10 were classified into the severe CSO-EPVS group (Figure 2A) and the remainder in the mild group (CSO-EPVS ≤10) (Figure 2B). As mentioned previously, a cutoff of EPVS >10 was used to represent moderate to severe EPVS (27).

### 2.6. Measurement and calculation of cerebral hemodynamic parameters

Patients completed hemodynamic monitoring in a constant temperature- and noise-controlled clinical investigation room. The participants were instructed to abstain from coffee, alcohol, and physical exercise for a minimum of 12 h and to abstain from chocolate and larger meals for a minimum of 4 h before the measurement. Cerebral hemodynamic monitoring was performed with blinding clinical information by a physician specializing in neurovascular ultrasound. In order to make sure that cerebral hemodynamic parameters were accurate, participants rested for 15 min while the equipment was put on.

Non-invasive continuous beat-to-beat blood pressure (NIBP) was recorded using a servo-controlled plethysmograph (Finometer, Finapres Medical Systems, Amsterdam, Netherlands) attached to the middle finger of the left hand, with a brachial cuff to correct B*p* values to the level of heart, and continuous cerebral blood velocity was recorded with transcranial Doppler ultrasound (MVU-6203, Delica Medical, ShenZhen, China) from the middle cerebral arteries (MCAs) using 2 MHz probes secured in place with a head-frame to maintain the optimal position and angle. The blood velocity of the middle cerebral artery (MCAv) signal was identified using established standards. The middle cerebral artery blood velocity signal was recognized by established criteria based on wave characteristics, signal depth, and the velocity constant throughout the testing session (28, 29). In the monitoring trend window, NIBP and flow velocity signals were displayed simultaneously at 125 Hz using the integrated hard disk of TCD, with waveforms continuously recorded for 15–30 min following stabilization.

A self-developed software processed the digital signals offline. We selected waveforms of NIBP and MCAv in 6 continuous cardiac cycles (that at least covered one respiratory cycle). The cross-correlation function was used to align pressure and flow velocity curves to remove possible time lags. Pulse pressure (PP) was computed as systolic blood pressure (SBP) minus diastolic blood pressure. The pulsatility index was calculated by PI = (Vs–Vd)/Vm. CCP and resistance area product (RAP) were calculated using the equation proposed by PANERAI (30).


$$CCP=\mathrm{MABP}-Vm*\left(\frac{\mathrm{MABP}-DBP}{Vm-Vd}\right)$$

$$\mathrm{RAP}=\frac{\mathrm{MABP}-DBP}{Vm-Vd}$$

The peak systolic velocity (Vs) was calculated as the mean of all peaks in the flow velocity curve corresponding to complete cardiac cycles. The diastolic velocity (Vd) was defined as the average of all troughs in the flow velocity curve corresponding to complete cardiac cycles. For each cycle, the mean blood velocity through the MCA (Vm) and mean arterial blood pressure (MABP) were determined. Vm was defined as the mean blood flow velocity over all complete cardiac cycles in the flow curve. The bilateral parameters were calculated independently, with data reported for the side with the higher CCP.

### 2.7. Statistical analysis

The Shapiro–Wilk test was performed to determine the normality of data distribution. Categorical variables were reported as the rate (percentage). Continuous variables with a normal distribution were characterized as mean ± standard deviation (SD), while those that were non-normally distributed were presented as the median (interquartile range). The independent sample t-test, Mann–Whitney U test, or chi-square test were used to compare the differences between the severe BG-EPVS group vs. the mild BG-EPVS group and the severe CSO-EPVS group vs. the mild CSO-EPVS group.

Spearman correlation analysis was performed to observe the correlation between hemodynamic parameters and EPVS. Grouping was considered as the dependent variable, and binary logistic regression analysis was performed to explore which hemodynamic parameter (including CCP, RAP, PI, and PP) was independently related to EPVS after adjusting for confounding factors.

Statistical analyses of the data were performed using SPSS software version 25.0 (SPSS, IBM). Two-tailed *p*-values <0.05 were considered as significant.

## 3. Results

### 3.1. Clinical characteristics of participants

A total of 125 participants without an acute cerebral event were enrolled; 107 participants completed the cerebral hemodynamics monitoring with complete and fully analyzable data. In this study, 11 participants were excluded because their imaging quality was insufficient (due to movement artifacts) to identify CSVD markers, and 7 people were excluded because they had poor Doppler envelopes.

Table 1 shows the clinical and demographic features of all subjects in both groups. The mean age was 63.8 ± 8.6 years, with 64 (59.8%) men. The severe BG-EPVS group had a greater proportion of men (73.7 vs. 44%, *p* = 0.002), a history of diabetes (33.3 vs. 24%, *p* = 0.039), and higher blood HbA1c levels (median: 5.90 vs. 5.65%, *p* = 0.003) compared with the mild group. There were no differences observed for the remaining clinical features. In terms of imaging characteristics, the group with severe BG-EPVS showed more severe WMHs [Fazekas score: 3 (2, 4) vs. 2 (1, 2), *p* = 0.001] and a greater proportion of lacune (57.9 vs. 30%, *p* = 0.004). This trend indicated that they are common imaging features of cerebral small vessel disease.

**Table 1:** General clinical characteristics of participants.

Characteristics	Overall	mild BG-EPVS group	severe BG-EPVS group	*p* value	mild CSO-EPVS group	severe CSO-EPVS group	*p* value
n	107	50	57	-	79	28	–
Age (years)	63.8 ± 8.6	62.10 ± 9.27	65.26 ± 7.83	0.058	62.32 ± 8.37	67.93 ± 8.16	0.003^**^
Male, n (%)	64 (59.8)	22 (44.0)	42 (73.7)	0.002^**^	43 (54.4)	21 (75.0)	0.056
BMI, kg/m^2^	23.91 ± 2.73	24.25 ± 2.94	23.62 ± 2.53	0.231	23.81 ± 2.67	24.22 ± 2.92	0.490
Smoking, n (%)	27 (25.2)	10 (20.0)	17 (29.8)	0.243	16 (20.3)	11 (39.3)	0.046^*^
Drinking, n (%)	6 (5.6)	3 (6.0)	3 (5.3)	0.869	5 (6.3)	1 (3.6)	0.586
Hypertension, n (%)	49 (45.8)	18 (36.0)	31 (54.4)	0.057	35 (44.3)	14 (50.0)	0.603
Diabetes, n (%)	27 (25.2)	8 (16.0)	19 (33.3)	0.039^*^	21 (26.6)	6 (21.4)	0.590
Cerebrovascular diseases, n (%)	31 (29)	12 (24.0)	19 (33.3)	0.288	21 (26.6)	10 (35.7)	0.360
Homocysteine, mmol/L	9.63 (8.28,12.3)	9.22 (7.92,11.99)	10.12 (8.39,12.46)	0.425	10.77 (8.85,10.77)	10.77 (9.03,11.02)	0.948
Fasting blood glucose, mmol/L	4.7 (4.31,5.4)	4.65 (4.31,5.2)	4.8 (4.3,5.74)	0.439	4.65 (4.30,5.25)	4.80 (4.33,5.72)	0.602
HbA1c, %	5.80 (5.40,6.50)	5.65 (5.30,6.20)	5.90 (5.60,6.75)	0.020^*^	5.80 (5.40,6.30)	5.90 (5.50,6.58)	0.779
Total cholesterol, mmol/L	4.5 ± 1.07	4.58 ± 0.8	4.42 ± 1.26	0.451	4.57 ± 1.09	4.30 ± 1.00	0.254
Triglyceride, mmol/L	1.23 (0.88,1.88)	1.32 (0.91,1.76)	1.08 (0.83,1.96)	0.886	1.33 (0.92,1.88)	0.96 (0.74,1.88)	0.113
Low-density lipoprotein, mmol/L	2.68 (2.19,3.2)	2.75 (2.3,3.06)	2.58 (1.73,3.27)	0.426	2.77 (2.20,3.23)	2.48 (1.93,3.03)	0.220
Hematokrit, %	39.66 ± 3.72	39.38 ± 3.56	39.9 ± 3.87	0.467	39.60 ± 3.76	39.83 ± 3.68	0.782
WMH, fazekas score	2 (1,3)	2 (1,2)	3 (2,4)	0.001^**^	2 (1,3)	3 (2,5.75)	0.001^**^
Lacunes, n (%)	48 (44.9)	15 (30)	33 (57.9)	0.004^**^	29 (36.7)	19 (67.9)	0.004^**^
EPVS, scale	3 (1,3)	1 (1,1)	3 (3,3)	<0.001^**^	1 (0,1)	3 (3,3)	<0.001^**^

BG, basal ganglia; EPVS, enlarged perivascular spaces; CSO, centrum semiovale; BMI, body mass index; HbA1c, hemoglobin A1c; WMH, white matter hyperintensities.

*p < 0.05; **p < 0.01.

The comparative results of general clinical characteristics between the severe CSO-EPVS group and the mild CSO-EPVS group were different from those between the severe BG-EPVS group and the mild BG-EPVS group. The age (mean: 67.93 vs. 62.32 years, *p* = 0.003) and the proportion of current smoking in the severe CSO-EPVS group were higher than in the mild CSO-EPVS group (39.3 vs. 20.3%, *p* = 0.046). There were no differences in the proportion of men, diabetes, and the levels of laboratory tests. However, imaging characteristics between the severe CSO-EPVS group and the mild CSO-EPVS group still showed the same trend with statistically significant differences [Fazekas score: 3 (2,5.75) vs. 2 (1, 3), *p* = 0.001; lacunes: 67.9 vs. 36.7%, *p* = 0.004].

### 3.2. The relationship between hemodynamic parameters and BG-EPVS

Table 2 shows the comparative results of the hemodynamic parameters including CCP, RAP, PI, PP, SBP, DBP, MABP, *Vs*, Vd, and Vm between the severe BG-EPVS group and the mild BG-EPVS. group.

**Table 2:** Cerebral hemodynamic parameters.

	mild BG-EPVS group	severe BG-EPVS group	*p* value	mild CSO-EPVS group	severe CSO-EPVS group	*p* value
CCP, mmHg	25.40 (19.85,30.7)	31.48 (26.41,36.17)	<0.001^**^	29.31 (22.24,32.98)	30.50 (24.07,37.08)	0.200
RAP	0.94 (0.80,1.10)	0.93 (0.81,1.11)	0.970	0.93 (0.81,1.09)	0.97 (0.79,1.23)	0.519
PI	0.86 (0.79,0.95)	0.91 (0.80,1.08)	0.142	0.86 (0.79,0.99)	0.90 (0.81,1.13)	0.519
PP, mmHg	50.59 (42.84,58.64)	48.95 (42.57,61.74)	0.755	50.14 (42.16,59.15)	49.20 (44.52,64.01)	0.457
SBP, mmHg	110.68 (101.5,126.67)	116.68 (107.66,132.54)	0.038^*^	112.17 (105.69,128.26)	117.78 (106.49,128.43)	0.387
DBP, mmHg	62.93 ± 9.70	67.64 ± 8.08	0.009^**^	65.26 ± 8.91	65.94 ± 9.91	0.736
MABP, mmHg	83.38 ± 12.25	88.39 ± 9.72	0.020^*^	85.57 ± 11.15	87.40 ± 11.46	0.462
*Vs*, cm/s	91.49 (78.08,107.4)	93.48 (75.79,108.94)	0.970	93.38 (80.35,109.33)	89.25 (72.77,106.86)	0.391
Vd, cm/s	39.38 (31.87,47.13)	38.79 (30.70,43.78)	0.428	39.43 (34.72,45.78)	32.46 (29.04,42.88)	0.052
Vm, cm/s	59.46 (51.94,70.37)	59.39 (48.30,70.76)	0.609	59.75 (52.98,69.95)	52.59 (46.36,70.95)	0.161

BG, basal ganglia; EPVS, enlarged perivascular spaces; CSO, centrum semiovale; CCP, critical closing pressure; RAP, resistance area product; PI, pulsatility index; PP, pulse pressure; SBP, systolic blood pressure; DBP, diastolic blood pressure; MABP, mean arterial blood pressure; Vs, peak blood velocity through the MCA; Vd, peak diastolic blood velocity through the MCA, Vm, mean blood velocity through the MCA.

*p < 0.05; **p < 0.01.

The severe BG-EPVS group had higher CCP (median: 31.48 vs. 25.40 mmHg, *p* < 0.001), SBP (median: 116.68 vs. 110.68 mmHg, *p* < 0.001), DBP (mean 67.64 vs. 62.93 mmHg), and MABP (mean: 88.39 vs. 83.38 mmHg). When BG-EPVS was used as a continuous variable, Spearman’s rank correlation coefficient with CCP was 0.331 (*p* < 0.001), 0.217 (*p* = 0.025) with PI, 0.239 (*p* = 0.013) with SBP, 0.213 (*p* = 0.028) with DBP, and was 0.223 (*p* = 0.021) with MABP, while no correlation was found with RAP or PP. When performing binary logistic regression analysis, SBP, DBP, and MABP were excluded to avoid subsequent collinear problems. The results of the binary logistic regression analysis showed that CCP was independently related to BG-EPVS after adjusting for sex, age, diabetes, HbA1c, WMHs, and lacunes. The detailed analysis results are shown in Table 3.

**Table 3:** Association between hemodynamic parameters and severe BG-EPVS.

Hemodynamic parameters	*B*	*p* value	*OR* (95%*CI*)
CCP, mmHg	0.076	0.027^*^	1.079 (1.009–1.155)
RAP	−0.369	0.703	0.692 (0.104–4.602)
PI	−1.535	0.434	0.215 (0.005–10.091)
PP, mmHg	−0.008	0.760	0.992 (0.942–1.044)

BG-EPVS, enlarged perivascular spaces in basal ganglia; CCP, critical closing pressure; RAP, resistance area product; PI, pulsatility index; PP, pulse pressure.

Multivariable logistic regression analysis was performed adjusting for sex, age, and significant (*p* < 0.05) variables from the univariate analyses (including sex, diabetes, HbA1c, WMHs, and lacunes). Severe BG-EPVS was defined as EPVS > 10 in basal ganglia.

*p < 0.05.

### 3.3. The relationship between hemodynamic parameters and CSO-EPVS

Table 2 shows the comparative results of the hemodynamic parameters including CCP, RAP, PI, PP, SBP, DBP, MABP, *Vs*, Vd, and Vm between the severe CSO-EPVS group and the mild CSO-EPVS group.

There was no statistical difference in hemodynamic parameters between the severe CSO-EPVS group and the mild CSO-EPVS group. When CSO-EPVS was used as a continuous variable, Spearman’s rank correlation coefficient with CCP was 0.193 (*p* = 0.047) and 0.215 (*p* = 0.026) with PI, while no correlation was found with RAP or PP. The results of the binary logistic regression analysis did not show any hemodynamic parameters independently associated with CSO-EPVS after adjusting for sex, age, smoking, WMHs, and lacunes. The detailed analysis results are shown in Table 4.

**Table 4:** Association between hemodynamic parameters and severe CSO-EPVS.

Hemodynamic parameters	*B*	*p* value	OR (95%*CI*)
CCP, mmHg	0.001	0.965	1.001 (0.939–1.068)
RAP	0.515	0.611	1.674 (0.231–12.155)
PI	−0.062	0.973	0.940 (0.025–35.085)
PP, mmHg	0.002	0.926	1.002 (0.953–1.054)

CSO-EPVS, enlarged perivascular spaces in centrum semiovale, CCP, critical closing pressure; RAP, resistance area product; PI, pulsatility index; PP, pulse pressure. Severe CSO-EPVS was defined as EPVS > 10 in centrum semiovale.

Multivariable logistic regression analysis was performed adjusting for sex, age, and significant (*p* < 0.05) variables from the univariate analyses (including age, smoking, WMHs, and lacunes).

## 4. Discussion

This study explored the association between cerebral hemodynamic parameters focused on CCP in BG-EPVS and CSO-EPVS, respectively. The binary logistic regression analysis showed that higher CCP, reflecting cerebrovascular tension, was independently correlated with severe BG-EPVS. However, it was not related to severe CSO-EPVS after adjustment for confounders. No further significant associations were found between EPVS and RAP, PI, and PP in either the BG or the CSO.

Numerous recent studies have underlined the importance of cerebral vascular hemodynamics in the pathophysiological mechanisms of EPVS, specifically PI (8–11), CVR (12, 13), and CBF (14). However, no studies to date have investigated associations between CCP and EPVS.

Although elucidation of the mechanism responsible for the relationship between higher CCP and BG-EPVS will require further examination in a properly designed study, the higher CCP reflects the change of cerebrovascular tension and is associated with impaired cerebral autoregulation (18). Regulation of vascular tone to blood pressure fluctuations is vital to maintain cerebral blood flow (31). Previous studies have identified CA as an important mechanism in the pathogenesis of cerebral small vessel disease (32, 33). It might be one of the reasons linking CCP and the development of EPVS. Elevated CCP might be a compensatory change in cerebral small vessels in response to hypertension. In our study, the blood pressure level was higher in the severe BG-EPVS group. Subjects with vascular risk factors (such as hypertension) tend to have EPVS in the BG and more severe WMH (34). One large prospective cohort study found high cumulative blood pressure exposure was an independent risk factor for EPVS (35). High vascular tone could affect cerebral circulation and indirectly impair cerebrospinal fluid flow, further contributing to the development of BG-EPVS (15, 36).

In contrast, no similar hemodynamic differences were found in the CSO-EPVS. This result may indicate that different regions of EPVS are involved in other pathophysiologic mechanisms. A previous study has shown an association between cerebral amyloid angiopathy (CAA) and CSO-EPVS (34). Another explanation could be the difference in feeding vessels, with the BG being supplied only by the MCA but the CSO also being provided by other major cerebral vessels. We measured the proximal MCA (M1 segment), which is closer in anatomical position to the supplying arteries (lenticulostriate arteries) of the basal ganglia region — however, the CSO is supplied with blood by the medullary artery of Duret.

## 5. Conclusion

This study identified an independent association between CCP and BG-EPVS and provides a new perspective for studies on the pathogenesis of EPVS.

## 6. Limitations

There are several limitations in this study. Firstly, the causal relationship between CCP and EPVS in basal ganglia could not be determined according to this observational study. It is needed for longitudinal research to further understand the correlations identified in our study. Secondly, the sample size of the severe CSO-EPVS group was relatively small (n = 28), which may have affected the statistical validity of the multivariate analyses. Thirdly, CCP cannot be evaluated directly because, in most clinical conditions, arterial blood pressure does not fall to the extremes that produce circulatory arrest. Prior studies relied on estimations derived from linear assumptions rather than real CCP (30). Future studies will require larger sample sizes to reduce data uncertainty and make more accurate models of the cerebrovascular bed.

## Data availability statement

The original contributions presented in the study are included in the article/supplementary material, further inquiries can be directed to the corresponding author.

## Ethics statement

The studies involving humans were approved by the Clinical Research and Application Ethics Committee of the Second Hospital of Guangzhou Medical University. The studies were conducted in accordance with the local legislation and institutional requirements. The participants provided their written informed consent to participate in this study. Written informed consent was obtained from the individual(s) for the publication of any potentially identifiable images or data included in this article.

## Author contributions

JZ and WL designed the study protocol and drafted the manuscript. JC was involved in data quality control and statistical analysis. JZ and JC were involved in patient collection. QG revised the manuscript, conceptualization, methodology, project management, and funding acquisition. All authors contributed to the article and approved the submitted version.
